# A Novel Approach to Transforming Smoking Cessation Practice for Pregnant Aboriginal Women and Girls Living in the Pilbara

**DOI:** 10.3390/healthcare6010010

**Published:** 2018-01-23

**Authors:** Paula Wyndow, Roz Walker, Tracy Reibel

**Affiliations:** Telethon Kids Institute, University of Western Australia, Perth 6872, WA, Australia; Roz.Walker@telethonkids.org.au (R.W.); Tracy.Reibel@telethonkids.org.au (T.R.)

**Keywords:** smoking, Aboriginal women, pregnancy, trauma, gender, culture, rural, remote, Western Australia

## Abstract

Tobacco smoking during pregnancy contributes to a range of adverse perinatal outcomes; but is a potentially modifiable behavior. In Australia Aboriginal and Torres Strait Islander women face a range of barriers that hinder; rather than support smoking cessation. Few smoking cessation programs consider the broader social determinants of women’s lives; the gendered nature of these or the complexities which impinge on behavior change in the presence of social and economic disadvantage and substantial individual and intergenerational trauma. Drawing on the salient gender and trauma-informed literature this paper describes the rationale underpinning formative research which will inform the design of a localized, culturally meaningful smoking cessation program for Aboriginal women living in the Hedland and Western Desert communities of the remote Pilbara region of Western Australia. We contend that a women-centered, trauma-informed approach to smoking cessation has much to offer those seeking to address this critical public health issue

## 1. Introduction

As a result of national, state and territory policies Australia has one of the lowest prevalence rates of smoking in the world [1]. Despite this achievement, Aboriginal and Torres Strait Islander people (herein referred to as Aboriginal people) are three times more likely to smoke tobacco than their non-Aboriginal counterparts [2]. In pregnancy this disparity is even larger with 48 percent of Aboriginal girls and women smoking, compared to 13 percent of their non-Aboriginal counterparts [3]. Aboriginal adolescents are three to four times more likely to smoke in pregnancy compared to non-Aboriginal peers [4] and recent data suggest an upwards smoking trend among this group in rural and remote areas [5].

Tobacco smoking during pregnancy is implicated in a range of adverse perinatal outcomes [6,7,8,9,10,11,12] but, it is a potentially modifiable health behavior. However, reducing the rates of Aboriginal women and girls smoking in pregnancy is a significant challenge when there are biological, (e.g., addiction [13]), historical, social and environmental factors that hinder, rather than support smoking cessation [14]. Despite a substantial body of literature describing why Aboriginal women and adolescent girls smoke, including social norms, addiction and as a means to cope with stress [14,15,16], there is limited evidence regarding the effectiveness of smoking cessation strategies and programs for Aboriginal women and girls during pregnancy [17,18]. Further, while there is recognition that Aboriginal women experience a range of adverse events including socioeconomic inequality and the ongoing effects of colonization, racism and the stolen generations [19,20], again few studies have sought to address these factors when designing smoking cessation programs for this target group. 

Thus, drawing on the available Australian and international literature of what has worked and what has not worked in supporting Indigenous women and girls to have smoke free pregnancies, this paper synthesizes the salient evidence to support the development of a novel approach to smoking cessation in a distinct geographical region. This includes describing the rationale for approaching the research from a women-centered and trauma-informed perspective from the outset and highlights the importance of drawing on local cultural knowledge to develop supportive approaches to smoking cessation more likely to gain traction with women in these communities.

## 2. A Novel Approach

A research project, Tackling Indigenous Smoking (TIS) in the Pilbara, is being conducted as a partnership between Rural Health West (a WA health workforce agency based in Perth, Australia), researchers from the Telethon Kids Institute and tobacco prevention officers working with Wirraka Maya Health Service Aboriginal Corporation (WMHSAC) in Hedland, Australia and Puntukurna Aboriginal Medical Service (PAMS) in Newman, Australia and surrounding remote communities, in the Pilbara region of Western Australia. The research is supported by an Australian Government Department of Health Tacking Indigenous Smoking (TIS) Innovation Grant. (More information can be found on the Australian Government Department of Health website at http://www.health.gov.au/internet/main/publishing.nsf/Content/indigenous-tis-innovation-grants-wa).

The key aims of TIS in the Pilbara are to:

(a) Explore the function of smoking in Aboriginal women lives and specific contextual issues that influence their smoking such as trauma and violence, disadvantage, identify formation and socials functions and stress;
(b) Design and deliver a relevant, culturally meaningful smoking cessation program for women living in the Hedland and Western Desert communities in the Pilbara region of Western Australia;
(c) Address Aboriginal women’s smoking in an integrated and holistic manner, as part of the health care they receive before, during and after pregnancy, within a primary health care setting; and,
(d) Enhance the evidence base on the effectiveness of a women-centered, trauma-informed approach to smoking cessation.

One of the key challenges in implementing programs or services designed to support positive changes in health behaviors is engaging with the population for whom a program is intended to benefit, in this case, Aboriginal women of childbearing age in a remote region of Western Australia. Thus, the formative stage of the research will rely on qualitative and Community Based Participatory Action Research (CBPAR) methods as a means of utilizing the key principles of a trauma—informed approach to establish a better understanding of the function of smoking in Aboriginal women lives who live in the specified towns and remote communities in the Pilbara. This approach includes: creating a culture of safety and trust; practicing cultural competence; supporting consumer control, choice and autonomy, genuine power sharing and governance; and, understanding that healing happens in relationships [21]. The potential benefits of using CBPAR and a trauma-informed approach are: the development of trust between researchers and the communities; the increased likelihood that the research will lead to programs that communities value and use; creating connections between community organizations, hospitals, clinics and researchers to share resources and knowledge; and, building on the strengths, skills and programs already in place in communities to reduce duplication of effort. 

## 3. Women-Centered Approaches to Smoking Cessation

A review of smoking cessation approaches (Expecting to Quit) found that strategies that include women-centered care show the most promise [22]. These strategies include comprehensive, holistic approaches that recognize the context of women’s lived experience, focus on women’s health before and after, not just during pregnancy and take into account the specific factors that contribute to women’s smoking such as trauma, violence, mental health and socioeconomic status. Unlike person-centered care which is gender neutral, women-centered care recognizes gender as a key determinant of women’s health [23] and seeks to empower women by addressing gender inequities that occur via gender norms, structures and relationships [24]. By building on women’s strengths, rather than deficits and enhancing women’s self-efficacy, this approach has the potential to transform both health and social outcomes for women [24]. 

Historically, smoking cessation health promotion activities have focused on individual behavior change without recognizing the gendered nature of smoking [25]. As more evidence about the immediate and long-term effects on children from maternal smoking emerged, the focus on quitting smoking in pregnancy has typically centered on the benefits for the baby, with less importance placed on the benefits for the mother [26]. For example, campaign slogans such as “Quit for New Life, Butt out for Bub”, while well intentioned, prioritized the needs of the fetus as primary, with the woman’s needs as secondary. By contrast, the Australian Government’s “Quit for you-Quit for two” campaign highlights the importance of women quitting for themselves, as well as for fetus and baby.

Adopting health promotion messages that shame and blame, rather than support or encourage women’s reduction or abstinence from smoking, may create more harm and stress for mothers [27]. Women who find it difficult to “quit for their baby” during pregnancy may feel they have failed and/or feelings of shame may be exacerbated. These types of messages may have also unintended negative consequences as they reduce, rather than increase the likelihood that women will present for antenatal care, especially early in pregnancy, for fear of being judged [28]. There is a need to move away from blaming individuals, to acknowledging, accepting and respecting individual capabilities, circumstances and culture as well as individual strengths which support women’s self-efficacy in changing their behavior [29]. Thus, reviewing current health promotion materials with service providers and Aboriginal women and girls about the messages they are conveying will be an important part of developing a supportive and culturally relevant program.

The literature emphasizes the importance of understanding how Aboriginal women’s identities, roles and relationships support or hinder their smoking cessation efforts. Work by Greaves [30] revealed the many ways in which Aboriginal women in Canada and Australia construct meaning around their smoking. These included: (1) organizing social relationships, e.g., using smoking to create some space or alone time, to facilitate social interactions, or conversely to create a barrier between self and others (protection); (2) creating an image, e.g., wanting to be seen as being ‘cool’; (3) controlling emotions, e.g., suppressing anger, or having something to do when bored; (4) exercising dependency, e.g., viewing a cigarette as a friend; and, (5) creating identity. Gould et al.’s exploration of Aboriginal women’s narratives about smoking [31] also revealed the significance of women’s relationships in their lives. Male partners and mothers were seen as having an important influence on women’s smoking, both as enablers of smoking or motivators for quitting. The start of a new relationship, especially with a non-smoker was found to be a motivator to quit, while being in a stressful relationship prompted women to smoke more [31].

For many women pregnancy is a catalyst for personal behavior change where they see themselves as protectors, or want to be good role models for their children [15,32]. Creating smoke free homes to protect the new baby from tobacco smoke appears to be something that pregnant women are motivated to do, although concerns about maintaining significant relationships can impede their success [16]. Pregnant women who have strong beliefs about the harm of smoking to children from second hand smoke (SHS) exposure are more likely to insist on smoke free homes [33], highlighting the importance of local, timely and explicit information, as well as skills to successfully negotiate smoke free areas in the light of resistance from partners and other family members. Thus, providing women with the support to create a smoke free home may be a critical ‘soft’ entry point for health professionals to start engaging with women around reducing or stopping smoking in pregnancy and this approach would need to be explored with health providers.

Finally, taking a woman-centered approach to smoking cessation, means addressing girls and women’s tobacco use across the life course. As well as during pregnancy, Gould et al.’s study [31] identifies key transition points in Aboriginal women’s lives when smoking interventions can occur. These include adolescence, turning 18 (legal age of smoking in Australia) and between pregnancies, i.e., when transitioning from breastfeeding to bottle or planning a subsequent pregnancy. As multiple attempts to quit smoking are often undertaken [34], recognizing other opportunities to provide support to women across their life course may not only lessen the possibility of women smoking during pregnancy but also prevent women from initiating smoking and/or relapsing after they have given birth.

## 4. Trauma and Smoking

Exploring the function of smoking in the lives of Aboriginal women and girls is an opportunity to understand why smoking among this cohort is so entrenched and how smoking cessation strategies might be included as part of a maternal model of care. This includes understanding the impact of trauma as a contributing factor to sustaining smoking in women’s lives. The US based Substance Abuse and Mental Health Services Administration (SAMHSA) publication “Concept of Trauma and Guidance for a Trauma-Informed Approach”, describes trauma as,

“an event, series of events, or set of circumstances that is experienced by an individual as physically or emotionally harmful or life threatening and that has lasting adverse effects on the individual’s functioning and mental, physical, social, emotional or spiritual wellbeing”.

[35] (p. 2)

The link between low socioeconomic status, stress and smoking is well established, as is the high rates of stress, mental health disorders and trauma that Aboriginal women and their families experience during pregnancy [15,36,37]. A South Australian study of Aboriginal women found that experiencing three or more social health issues in pregnancy was associated with smoking cigarettes and being at a greater risk of psychological distress postpartum [37]. A similar study in New South Wales found that about forty per cent of pregnant Aboriginal women exhibited symptoms of Post-Traumatic Stress Disorder (PTSD), with just under half reporting at least one death of a close friend or family member [36]. Pregnant Aboriginal women in Western Australia reported that stopping smoking in pregnancy was not a priority for them, given the socioeconomic challenges they were experiencing [15]. For Aboriginal women then, the intergenerational effects of colonization, dispossession and racial discrimination, all of which involve some degree of trauma, in addition to other traumatic life events are all contributing factors to Aboriginal women’s smoking behaviors [19,20,38].

## 5. Trauma Informed Care and Practice

For health providers, the practical application of a trauma-informed approach when seeking solutions to supporting women to stop smoking requires an understanding of how women may be using smoking as a coping strategy, or as an adaptive response that has likely worked for them in the past. Rather than focusing on what is ‘wrong’ with a woman, the trauma-informed approach is to ask “what has happened to this woman?” that has brought them to this point. This places the smoking behavior within the context of the woman’s life and underpins the relational nature of health providers supporting women to create self-efficacy [39]. We propose that trauma-informed approaches to smoking cessation interventions require health professionals being aware of the impact of trauma in women’s lives and taking care to avoid judgmental or shaming approaches when addressing women’s smoking, as this is likely to cause further harm and/or create barriers for women who want help to stop smoking [40].

Furthermore, the overall culture of the health service organization plays a critical role in supporting women in a way that minimizes the risk of triggering further trauma and fostering a reluctance to seek help. For instance, whilst a **trauma specific service** provides specialized or therapeutic treatment for current or past trauma, a **trauma-informed service** is not dependent on disclosure of trauma by individuals. Instead, the framework through which a service responds to their clients is trauma-informed, with the lens and language used by all staff focused on supporting all people using the service to feel valued as individuals, as well as safe and empowered [40]. Hopper, Bassuk & Olivet, define trauma-informed care as:

“a strengths-based framework that is grounded in an understanding of and responsiveness to the impact of trauma, that emphasizes physical, psychological and emotional safety for both providers and survivors and that creates opportunities for survivors to rebuild a sense of control and empowerment”.

[41] (p. 82)

The Mental Health Coordinating Council, the peak body for community mental health organizations in New South Wales, following a synthesis and adaption of trauma-informed approaches in the literature have proposed eight core principles to underpin trauma-informed care and practice (TICP) for organizations wanting to reframe their service delivery [21]. These include: (1) understanding trauma and its impact; (2) creating a culture of safety and trustworthiness; (3) ensuring cultural competence; (4) supporting consumer control, choice and autonomy; (5) sharing power and governance; (6) integrating care; (7) understanding that healing happens in relationships; and, (8) believing that recovery from trauma is possible. Becoming a trauma-informed organization therefore requires a shift in organizational culture, practice and theoretical framing [42], with a commitment to regularly reviewing policies and practices in order that services minimize the risk of triggering further trauma and a reluctance to seek help [43].

The TICP core principles are complemented by the nine principles and priorities used for both the 2013–2023 National Aboriginal and Torres Strait Islander Health Plan (NATSIHP) and the 2012 National Tobacco Strategy, described in a recently published systematic review [18]. The review synthesized the evidence about reducing smoking amongst Indigenous people, describing the centrality of: equality, partnership, engagement, social and emotional wellbeing, cultural respect, human and community capability, accountability and evidence based practice; within a whole of life context. The complementarity across ‘best practice’ principles in the literature highlights the need for organizations to be mindful of the impact of a service environment on individuals and communities and avoid conditions which may alienate women from seeking assistance. 

In respect to the clinical relationship supporting self-efficacy, Reeves review of the literature on trauma-informed care [44] highlights the potential for power imbalances between health professionals and the client/patient, if the provider positions themselves as the expert. In such a scenario, the skills and strengths of the patient/client are not recognized or utilized. Instead, working in a genuinely collaborative way with clients from the outset to co-design a smoking cessation program or intervention that has meaning for them and provides choices, will help to break down the idea of health professionals as experts and provide a space for the client’s knowledge and skills to be heard and guide the process. Achieving trust and safety in the provider/client relationship is something that must be earned and demonstrated over time for it to be effective [45]. As such, we view this element of a trauma-informed approach as essential in creating the circumstances for change to occur. 

## 6. Cultural Competence in a Trauma Informed Organization

Taking into account the evidence related to women-centered and trauma-informed approaches to smoking cessation, the formative aspect of this research will also explore health professionals understanding of trauma as a contributing factor to smoking behaviors within the context of women’s social, familial and cultural circumstances in the Pilbara region. This includes identifying the challenges that health providers may face in incorporating trauma-informed care and practice into their professional relationships with women in the clinical setting, particularly if health professionals have limited understanding of the local cultural contexts in which they are working. To address this, Walker, Schultz and Sonn [46] emphasize the critical importance of cultural competence in transforming policies and clinical practice aimed at improving the physical health, mental health and social and emotional wellbeing of Aboriginal people.

Cultural competence is “a set of behaviors or policies existing in an organization or other group of individuals that permits the provision of effective services in cross cultural situations” (Cross, Bazron Dennis & Isaacs 1989 cited in Walker et al. 2014 [46]). In the Australian context, cultural competence also requires a commitment to gender-informed and trauma-informed services, policies, programs and practice. This approach is crucial in achieving service delivery models which support families, especially women, during the perinatal period [47,48].

## 7. Intersection of Gender, Trauma and Culture

Currently, examples of initiatives in which Indigenous approaches and the role of culture are integrated into women-centered, trauma-informed approaches are scarce in the Australian Aboriginal and Torres Strait Islander context. Programs generally tend to focus on only one or two elements of women’s wellbeing and few have explicitly and/or effectively embraced the intersection of culture, gender and trauma to support recovery, such as has been implemented in the Canadian context. For example, the trauma-informed Seeking Safety counselling model, an intervention for women with both substance abuse disorder and trauma [49] when delivered together with traditional healing methods showed a strong and positive impact on women’s experience of intergenerational trauma and substance use disorder [50,51]. Similarly, a heart health pilot among Indigenous women in Canada has also shown some promise [52], integrating Indigenous knowledge into more mainstream health promotion messaging, with participating women reporting small but positive changes to their physical, emotional and spiritual health. By being encouraged to be on their own health journey, the women were supported to make changes in their lives. Both these programs used a talking circle as a means to share information among participants, thus contributing to women’s self-efficacy premised on collective knowledge sharing [53]. As such, this is a culturally relevant approach to consider for Aboriginal women in WA, who also use collective knowledge sharing as a usual practice in daily living and for who sharing stories is an important aspect of knowledge building.

In Australia, the Talking About the Smokes (TATS) project provides further salient information about addressing smoking in Aboriginal communities. In this national study, 34 Aboriginal Controlled Community Health Services (ACCHS) reported that the risks associated with smoking were well known among participants [54]. It found that most Aboriginal people who smoked wanted to quit [55], seventy per cent had tried previously to quit, although those living in the most disadvantaged areas had less desire to quit [56]. This is useful information for health professionals who may either consciously or unconsciously assume that Aboriginal women do not want to stop smoking. In an applied sense, instead of directly asking women whether they want to stop smoking, it is more beneficial in the health professional and patient/client relationship to instead understand what knowledge a woman has about the health effects of smoking, if there have been previous quit attempts and what, if any, support she has received in the past. This approach builds a more accurate picture of a woman’s smoking story. For example, in a study chronicling smoking initiation and history, Aboriginal women expressed intense regret about smoking in pregnancy and wished they had received more support to quit at that time [31].

Returning again to the international literature, a systematic review addressing Canadian Indigenous women’s perspectives of maternal health care services [57] considered the policy environment which interrupts women’s access to appropriate health care, including access to smoking cessation programs. The review found that inconsistent advice provided to pregnant women who smoked, as well as factors such as substance abuse, unstable domestic situations and low socio-economic circumstances compromised the ability of women to attend cessation programs. Thus, in order to promote better maternal health a more consistent and cohesive approach to providing Indigenous women with the support they need across a range of health behaviors including smoking, is required. Further, a 2013 Canadian study which included a significant number of Aboriginal women (~40%) also identified the need for multifaceted, comprehensive and tailored approaches, with multiple entry points for pregnant women to access smoking cessation programs, if these were to succeed [58]. The study also highlighted the importance of engaging local women in program development to promote ownership and uptake of cessation opportunities in the local community.

Finally, the provision of culturally safe services has been highlighted in the Australian context as a key factor in supporting good outcomes for Aboriginal mothers [59], as well as repeated calls for integrated service provision that address a range of social factors such as housing, employment, family and domestic violence, mental health and multi-substance abuse. For example, research by Passey, Sanson-Fisher, D’Este, & Stirling, [60] found that women using one substance in pregnancy were more likely to be using others, thus in addressing smoking, women’s alcohol and other drug use also needs to be considered. Subsequent follow up of the participants in the TATS program found that becoming employed and getting a house were key factors that precipitated a quit attempt or were associated with stopping smoking for at least one month [61]. Taken together, these findings support the view that programs aimed at reducing or stopping smoking in Aboriginal communities need to take into consideration the socio-cultural determinants of health and move beyond addressing smoking in isolation.

When taken as a whole, the Australian and international research which refers to Indigenous populations and reports on smoking policy and cessation practices clearly shows there are considerable barriers to overcome when approaching smoking cessation intervention activities, as tobacco smoking does not exist in isolation but is deeply connected to a range of social and cultural determinants as well as other health behaviors. It is evident that reducing Aboriginal women’s smoking requires consideration of the intersection of culture, gender and trauma and how the interplay of these impact on conditions which are conducive to enabling women to think differently about the function of smoking in their lives. When this is more fully understood and acknowledged by health professionals, it will be possible to identify which support mechanisms Aboriginal women require to make significant and long lasting changes.

## 8. Conclusions and Next Steps

This paper has synthesized the salient gender and trauma-informed literature which supports an innovative approach to building self–efficacy among Aboriginal women and their families and the health professionals who support them during pregnancy and the postnatal period. We have hypothesized that using a women-centered, trauma-informed lens and a Community Based Participatory Action Research (CBPAR) approach will enable us to work together with participants in the Pilbara region to draw on individual and community knowledges and understandings about the reasons for and consequences of, tobacco smoking; as well as more culturally responsive ways of slowing down or quitting smoking.

We contend that this process will contribute to identifying how health services and health practitioners are better able to address this complex health behavior, using supportive measures which draw on women’s strengths and capacity for positive change. This is likely to include organizational changes which support a trauma- and gender-informed approach to service provision and increasing the cultural competence of health professionals working in the service environment. Further, using CPAR with women, their families and communities will help facilitate a sense of individual and collective efficacy and empowerment essential to reduce the rates of smoking. A more comprehensive report of the methods and outcomes associated with creating this novel approach to smoking cessation will be fully described in future publications.
